# Telocytes play a key role in prostate tissue organisation during the gland morphogenesis

**DOI:** 10.1111/jcmm.13234

**Published:** 2017-08-24

**Authors:** Bruno D. A. Sanches, Juliana S. Maldarine, Bruno C. Zani, Guilherme H. Tamarindo, Manoel F. Biancardi, Fernanda C. A. Santos, Paula Rahal, Rejane M. Góes, Sérgio L. Felisbino, Patricia S. L. Vilamaior, Sebastião R. Taboga

**Affiliations:** ^1^ Department of Structural and Functional Biology State University of Campinas Campinas São Paulo Brazil; ^2^ Department of Biology Univ. Estadual Paulista (UNESP) São José do Rio Preto São Paulo Brazil; ^3^ Department of Histology Embryology and Cell Biology Federal University of Goiás Goiânia Goiás Brazil; ^4^ Department of Morphology Institute of Biology (IB) Univ. Estadual Paulista – UNESP Botucatu São Paulo Brazil

**Keywords:** telocytes, CD34, c‐Kit, tissue remodelling, ventral prostate, morphogenesis, smooth muscle cell, TGF‐β1

## Abstract

Telocytes are CD34‐positive interstitial cells, known to exert several functions, one of which is a role in tissue organisation, previously demonstrated by telocytes in the myocardium. The existence of telocytes in the prostate has recently been reported, however, there is a lack of information regarding the function of these cells in prostate tissue, and information regarding the possible role of these cells in prostatic development. This study used immunofluorescence techniques in prostate tissue and prostatic telocytes in culture to determine the relationship between telocytes and prostate morphogenesis. Furthermore, immunofluorescent labelling of telocytes was performed on prostate tissue at different stages of early postnatal development. Initially, CD34‐positive cells are found at the periphery of the developing alveoli, later in the same region, c‐kit‐positive cells and cells positive for both factors are verified and CD34‐positive cells were predominantly observed in the interalveolar stroma and the region surrounding the periductal smooth muscle. Fluorescence assays also demonstrated that telocytes secrete TGF‐β1 and are ER‐Beta (ERβ) positive. The results suggest that telocytes play a changing role during development, initially supporting the differentiation of periductal and perialveolar smooth muscle, and later, producing dense networks that separate alveoli groups and form a barrier between the interalveolar region and periurethral smooth muscle. We conclude that telocytes play a relevant role in prostate tissue organisation during postnatal development.

## Introduction

Prostate development occurs as a result of a complex network of interactions between different molecular signalling pathways. These interactions initially occur between the epithelium of the urogenital sinus (UGE) and mesenchymal urogenital sinus (UGM), leading to the formation of epithelial buds derived from the UGE that invade the UGM. Prostate development begins with the epithelial–mesenchymal interaction of these buds with peripheral condensed mesenchyme, leading to branching, followed by differentiation of the proximal portions of the branches to form the conductive structures of the gland, the prostatic ducts and the distal portions that form the secretory structures, the prostate alveoli 1, 2, 3, 4, 5. The occurrence of such molecular interactions are spatially compartmentalised between different cell types involved in prostate development, such as the progenitor cells of mesenchymal fibroblasts, smooth muscle and basal epithelial cells 6, 7, 8.

The recently characterised telocytes (TCs) 9 are stromal cells present in various tissues 10, 11, 12, 13, 14, 15, 16, 17, exhibiting a reduced cell body and carrying long cytoplasmic projections known as telopodes. Telepodes can be divided into dilated portions, podoms and fibrillar‐like sections called podomers 8, 9. Several functions of telocytes have been proposed, which vary from organ to organ, such as organisation of the stroma through modulation of intercellular communication 15, regeneration of cardiac muscle 10, immune response in the duodenum 11, contractility of the uterus 12 and in intercellular electrical communication 18, among others.

Telocytes differ from other interstitial cells, such as interstitial Cajal cells (ICCs) by their characteristic morphology mentioned above, in addition to the fact that they are CD34‐positive cells. ICCs, described in organs of the digestive system, such as the stomach and intestine, possess pacemaker activity *via* the c‐kit receptor 19, 20, 21, the ligand of which is the stem cell factor (SCF), that acts in a way to facilitate calcium mobilization *via* a src family kinase and PI3K (Phosphatidylinositol‐4,5‐bisphosphate 3‐kinase) 22. Telocytes may also exercise pacemaker function by means of the c‐Kit, but may also possess a supportive function for the contraction of periductal smooth muscle, possibly by means of c‐kit‐independent pathways, such as *via* the SK3 (small conductance calcium‐activated potassium Channel 3) pathway 20, 23. The function of telocytes varies between organs 8, 10, 11, 12, 17, 18, 24, 25, and their role in the prostate remains elusive, in addition to lack of information regarding the development of these cells in prostate tissue.

This study aimed to evaluate the presence of telocytes during prostate development, and the possible role of telocytes on prostate morphogenesis and tissue organisation in the Mongolian gerbil. This species of rodent is a promising model for studies involving the prostate, as unlike other laboratory rodents, a functional prostate is present in females. Furthermore, this species has greater sensitivity to hormone treatments 4, 26, 27, 28, 29. For this purpose, immunofluorescence assays were performed on prostatic tissue, in addition to cultured prostatic cells.

## Materials and methods

### Animals and experimental design

The animals were provided by São Paulo State University (UNESP, São José do Rio Preto). Gerbils were housed in a temperature‐controlled (25°C) room on a 12 hrs light/dark cycle. All animals were housed in polyethylene cages, with *ad libitum* access to filtered water and rodent food. Animal handling and experiments were performed in accordance with the ethical guidelines of UNESP (ethics committee number 115/2015 CEUA). Ten adult female and 10 adult male gerbils (*Meriones unguiculatus*, Muridae: Gerbillinae), aged between 3 and 4 months, were mated. One male and one female were randomly matched to form independent litters. After birth, pups were split into five groups, four of which were used for immunofluorescence and ultrastructural analyses of postnatal prostate development in the gerbil at postnatal day 1 (P1), P7 (postnatal day 7), P14, P30 and P45 (*n* = 4 males in each group), whereas the sixth group (*n* = 5 males) formed the group killed at P120 for establishment of the telocyte primary cell culture. All animals were killed by lethal injection of a mixture containing ketamine as an anaesthetic (100 mg/kg body weight; Dopalen, Vetbrands, Jacareí, Brazil) and xilazine as a muscle relaxant (11 mg/kg body weight; Rompun, Bayer, Brazil).

### Isolation and primary culture of telocytes from prostate tissue

The isolation and primary culture of TCs from prostate tissue were performed following the protocol described by Bei *et al*. 30. After gerbils were killed, the prostate was dissected under sterile conditions and maintained in Hanks' balanced salt solution (HBSS, R21–022‐CV; Corning, Corning, NY,USA) supplemented with 100 U/ml penicillin, 100 μg/ml streptomycin (R30–002‐CI; Corning) and 0.01 mM HEPES(H3375; Sigma Aldrich, St.Louis, MO,USA). After being transported to the cell culture room and rinsed again with fresh HBSS, the prostates were transferred into a sterile culture dish containing Dubelcco's modified eagle medium nutrient mixture F12 (DMEM/F12; Sigma‐Aldrich) supplemented with 0.25 mg/ml collagenase type II (17101–015; Invitrogen, Paisley, Renfrewshire,UK). The prostates were minced into 1 mm^3^ pieces, transferred into a 50 ml centrifuge tube, then incubated on an orbital shaker at 37°C for 35 min. with the collagenase solution described above. Following the incubation, 25 ml of ice‐cold HBSS was added into the digest to inhibit collagenase activity. The dispersed cells were separated from the non‐digested tissue bypassing the mixture through a 40‐μm diameter cell strainer, then cells were collected by centrifugation at 1000 r.p.m. for 5 min. at 4°C. The cells were washed once with HBSS, centrifuged, then resuspended in a solution containing 10 ml DMEM/F12 supplemented with 10% foetal bovine serum (FBS, 16000–044,Gibco, Waltham, MA, USA),100 U/ml penicillin and 100 μg/ml streptomycin. Cells were seeded into sterile culture dishes and cultured in a humidified atmosphere of 5% CO2 at 37°C for 2 hrs to allow fibroblast attachment, in order to separate the fibroblasts from telocytes, as they adhere primarily to culture dish. Later, the unattached cells (containing TCs) were collected and cultured in DMEM/F12 supplemented with 10% FBS, 100 U/ml penicillin and 100 μg/ml streptomycin for 24 hrs, after which the culture medium was changed. Cell cultures were examined using an inverted biological microscope (IX50; Olympus,Tokyo, Japan) and TCs were photographed under 200× magnification 48 and 96 hrs after seeding.

### Ultrastructural analysis

Ultrastructural analysis was performed using the protocol described by Corradi *et al*. 8. Fragments of the developing prostate of gerbils at three different ages (P1, P7 and P45) were minced into small pieces and fixed by immersion in 3% glutaraldehyde plus 0.25% tannic acid solution in Millonig's buffer, pH 7.3, containing 0.54% glucose for 24 hrs. After washing with the same buffer, samples were post‐fixed with 1% osmium tetroxide for 1 hr, washed in buffer, dehydrated in a graded acetone series and embedded in Araldite resin. Ultrathin sections (50–75 nm) were prepared using a diamond knife and stained with 2% alcoholic uranyl acetate for 30 min. followed by 2% lead citrate in a 1 M sodium hydroxide solution for 10 min. Samples were evaluated by electron microscopy using a LEO – Zeiss 906 TEM at 80 kV.

### Immunofluorescence of paraffin‐embedded tissue sections

Immunofluorescence was performed on paraffin‐embedded tissue sections using the protocol described by Lima *et al*. 31. The prostate samples were fixed in 4% paraformaldehyde (buffered in 0.1 M phosphate, pH 7.4) for 24 hrs. After fixing, the tissues were washed in water, dehydrated in a series of ethanol solutions, embedded in paraffin (Histosec; Merck, Darmstadt, Germany),then sectioned at 5 μm using a microtome (RM2155, Leica, Nussloch, Germany). In order to verify the presence of telocytes during the differentiation of smooth muscle cells, tissue sections were subjected to double immunofluorescence assays for CD34/CD31 (mouse polyclonal CD34 IgG, B‐6, sc74499; rabbit polyclonal CD31 IgG, M‐185, sc‐28188; Santa Cruz Biotechnology, Dallas, TX, USA), CD34/c‐Kit (mouse polyclonal CD34 IgG, B‐6, sc74499; rabbit polyclonal c‐Kit IgG, C‐19, sc168; Santa Cruz Biotechnology) and CD34/TGF‐β1 (mouse polyclonal CD34 IgG; rabbit polyclonal TGF‐β1 IgG, V, sc146; Santa Cruz Biotechnology), which were incubated overnight at a dilution of 1:100. The next morning, sections were incubated with goat antimouse FITC‐labelled (sc‐2011; Santa Cruz Biotechnology) and goat anti‐rabbit Texas Red‐labelled (sc‐2780; Santa Cruz Biotechnology) secondary antibodies, diluted 1:200 in 1% bovine serum albumin (BSA) for 2 hrs at room temperature, then stained with DAPI (F36924; Life Technology, Grand Island, NY, USA). The histological sections were analysed with a ZeissImager M2 fluorescence microscope (Zeiss, Oberkochen, Germany) coupled to AxioVision (Zeiss) software.

### Immunofluorescence for telocyte cell culture

Immunofluorescence of the telocyte cell cultures was performed using the protocol described by Bei *et al*. 30. After three washes with PBS, telocytes were fixed in 4% paraformaldehyde for 30 min., washed with PBS, then permeabilised with 0.5% TritonX‐100 for 30 min. Another wash with PBS was performed, after which cells were blocked with 3% BSA for 1 hr. After blocking, cells were incubated overnight at 4°C with ERβ (rabbit polyclonal IgG, H‐150, sc‐8974; Santa Cruz Biotechnology), to evaluate whether this receptor would be expressed in prostatic telocytes, considering the importance of this receptor for late postnatal prostate development [5, 32, 33] and CD34 (polyclonal mouse, IgG, B‐6, sc74499; Santa Cruz Biotechnology), which is the major marker for telocytes. Primary antibodies were diluted 1:100 in 1% BSA, after washing three times with PBS, cells were incubated with goat antimouse FITC‐labelled (sc‐2011; Santa Cruz Biotechnology) and goat anti‐rabbit Texas Red‐labelled (sc‐2780; Santa Cruz Biotechnology) secondary antibodies diluted 1:200 in 3% BSA for 2 hrs, then stained with DAPI (F36924; Life Technology). Cells were kept in fresh PBS in the dark at 4°C prior to observation. Images were taken under 200× magnification with a fluorescent inverted microscope (DMI4000 B;Leica). Similar procedures were used for the double immunofluorescence staining for CD34 (mouse polyclonal IgG; Santa Cruz Biotechnology) and TGF‐β1 (rabbit polyclonal IgG; Santa Cruz Biotechnology).

## Results

### Light microscopy

**Figure 1:**
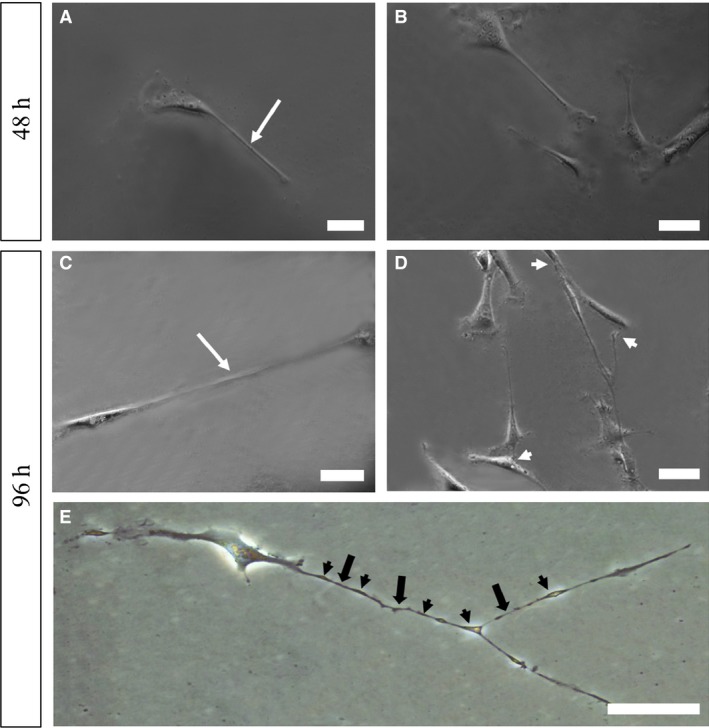
Phase‐contrast microscopy images of prostatic telocytes in primary culture. (**A**) Prostate telocyte isolated after 48 hrs in primary culture. (**B**) Formation of a network of telopodes by telocytes after 48 hrs in primary culture. (**C**) Prostate telocyte isolated after 96 hrs in primary culture, for which a long telopode can be observed. (**D**) Formation of a network of telopodes by telocytes after 96 hrs in culture.(**E**) Inverted light microscopy image of a telocyte in cell culture, in which the monoliform aspect of telopodes, alternating between podomers (fibrillar‐like segments) and podoms (dilated regions) is verified. White arrows (telopodes), white arrowhead (connections between telopodes); black arrow (podomers), black arrowhead (podoms). Original magnification of 200×, scale bars represent 50 μm.

Phase‐contrast microscopy showed the presence of telocytes after 48 hrs in primary culture, with shorter telopodes (Fig. 1A). The formation of telopode networks began after this period (Fig. 1B). Following 96 hrs of primary culture, telocytes showed long telopodes (Fig. 1C), and a telopode network was observed (Fig. 1D) as the monoliform aspect of the telopodes (Fig. 1E).

### Ultrastructural analysis

**Figure 2:**
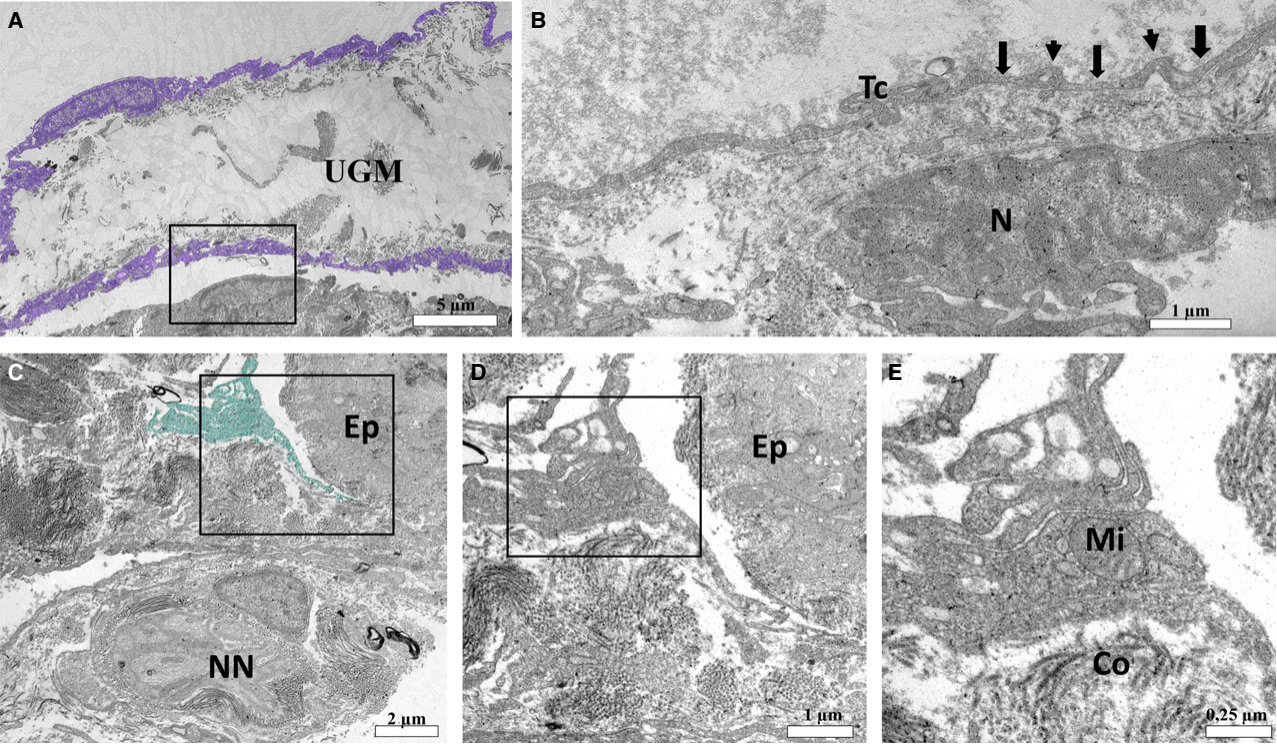
Ultrastructure of the developing prostate of neonates (on P1)**.** (**A**) Telocyte observed in prostatic mesenchyme on P1 with a long telopode (digitally coloured in blue). (**B**) Detail of a telocyte in the periphery of the prostatic mesenchyme in which the monoliform aspect of the telopode is observed, alternating between the podomers (thin fibrillar‐like segments) (arrow) and podoms (dilated, cisternae‐like regions) (arrowhead). (**C**–**E**) At the periphery of a prostatic budding, there are large cytoplasmic processes of an interstitial cell in which mitochondria can be observed. UGM (urogenital sinus mesenchyme), Tc (telopode), N (nucleus), NN (nerve), Ep (epithelium), Mi (mitochondria), Co (collagen fibrils).

**Figure 3:**
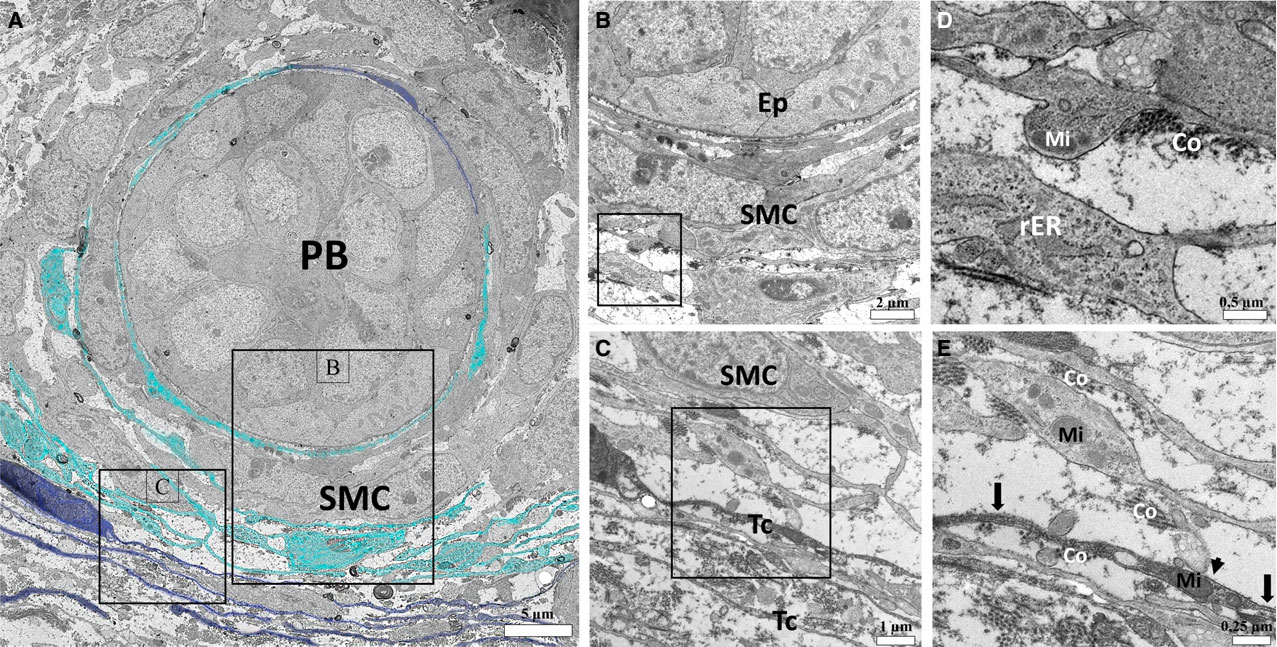
Ultrastructure of the developing prostate on early postnatal development (on P7). (**A**) It is possible to observe an intricate network of thick cell extensions that is interspersed with the developing periductal smooth muscle (digitally stained in cyan), external to this, it can observed telocytes, with their extremely thin telopodes (digitally stained in dark blue). (**B**) Detail of a cell with a pyramidal cell body with large cytoplasmic processes in the surrounding region of the periductal smooth muscle. (**C**) Detail of a telocyte at the periphery of these cells with thick cytoplasmic processes surrounding a smooth muscle cell. (**D**) Detail of thick cytoplasmic processes around the periductal smooth muscle, with a dilated rough endoplasmic reticulum, around them it is possible to observe a large deposition of collagen fibrils. (**E**) Detail of a thick cytoplasmic process next to a telopode, in which mitochondria is verified. In the telopode it can be observed the alternation of fibrillar‐like segments (podomers) and dilated regions (podoms). (PB) prostatic budding, SMC (smooth muscle cell), Ep (epithelium), Tc (telocyte), Mi (mitochondria), rER (rough endoplasmic reticulum), arrows (podomers), arrowhead (podoms).

**Figure 4:**
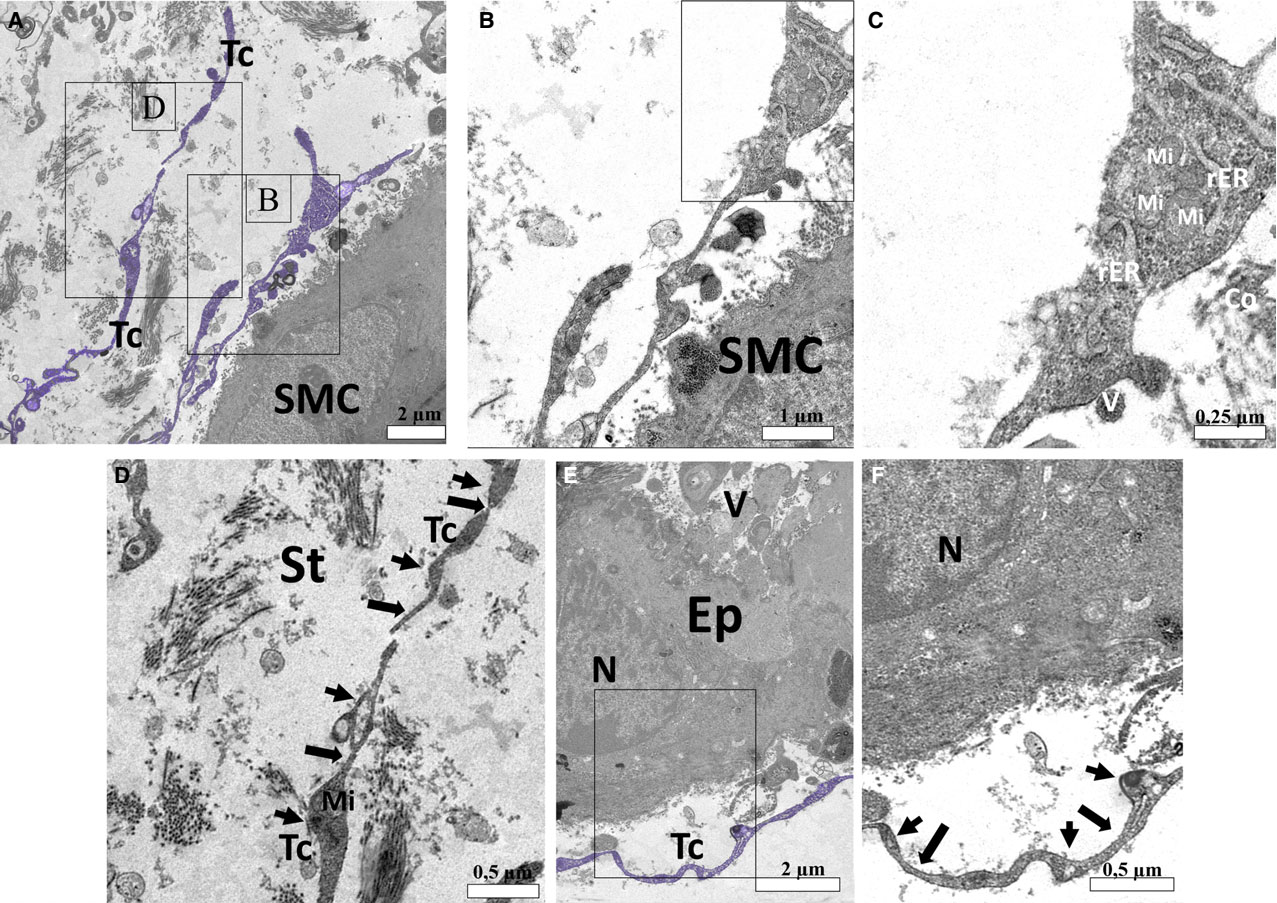
Ultrastructure of the developing prostate on gland prematuration (on P45). (**A**) Telocytes can be observed in the prostatic stroma at the periphery of a smooth muscle cell. A cell with a triangular cell body and cytoplasmic extensions can be observed. (**B**) Detail of this cell at the periphery of the periductal smooth muscle. (**C**) In larger detail, a broad amount of mitochondria and rough endoplasmic reticulum can be observed, in addition to vesicles. Surrounding this cell, collagen fibrils are verified. (**D**) Detail of telocytes in the prostatic stroma, in which telopodes of monoliform aspect can be observed, with the alternation between podomers and podoms. (**E**) Telocyte observed surrounding a prostatic alveolus. (**F**) The alternation between podomers (fibrilar‐like sections) and podoms (dilatations) in the telopode is also verified. Tc (telocyte); SMC (smooth muscle cell); Mi (mitochondria), rER (rough endoplasmic reticulum); V (vesicle); Co (collagen Fibrils); St (stromal); N (nucleus); Ep (epithelium); Seta (podomer); arrowhead (Podom).

The ultrastructure of the prostate of the neonates on P1 did not attest to the existence of telocytes in the peripheral region of the developing prostate budding, in this region cells with large cytoplasmic processes are verified, in which mitochondria can be seen (Fig. 2 C–E). Differentiated telocytes with their telopodes of monoliform aspect alternating between thin fibrillar‐like segments (arrows) and podoms (dilated, cisternae‐like regions) are observed in the interstitium (Fig. 2A and B). Later in the prostatic development, on P7, an intricate network of thick cytoplasmic processes that is interspersed with the developing smooth muscle cells can be observed, external to this, telocytes are verified, with their extremely thin telopodes that are arranged in a way to involve the developing smooth muscle layer (Fig. 3A, C, E). A cell with a pyramidal cell body with thick cytoplasmic processes surrounding periductal smooth muscle can be observed. In the cytoplasmic processes, mitochondria and dilated rough endoplasmic reticulum are verified, around these cytoplasmic processes it is possible to observe the large deposition of collagen fibrils (Fig. 3B and D), such cells may constitute differentiating telocytes. Later, differentiated telocytes can be observed at the periphery of the prostatic alveoli, surrounding the differentiated periductal musculature (Fig. 4E and F), telocytes are also observed forming a network of telopodes in the prostate interstitium (Fig. 4A and D). In the periphery of some alveoli can also be seen cells similar to the telocytes with triangular cell body and cytoplasmic processes, such processes are thick and carry mitochondria and rough endoplasmic reticulum, in addition to vesicles (Fig. 4B and C).

### Immunofluorescence analysis

**Figure 5:**
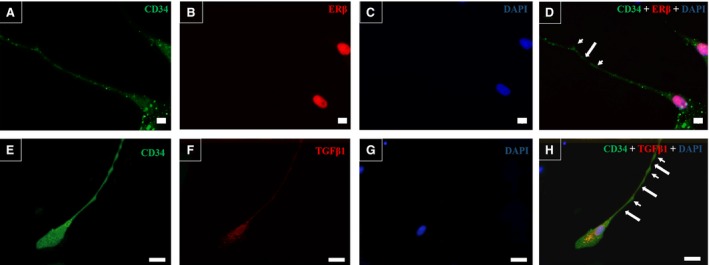
Double immunofluorescence assays for CD34/ERβ and CD34/TGF‐β1 in telocytes after 96 hrs in primary culture. (**A**) Image from the inverted fluorescence microscope showing CD34 labelling (green) in telocytes. (**B**) The nucleus of telocytes was positive for ERβ (red). (**C**) Marking of nuclei with DAPI (blue). (**D**) Overlay demonstrating the colocalisation of ERβ and DAPI labelling for the telocyte nuclei (purple) and cellular surface labelled with CD34 (green). (**E**) CD34 labelling (green) throughout the cell surface of telocytes. (**F**) Labelling of TGF‐β1 (red) throughout the telocytes. (**G**) Nuclei stained with DAPI (blue). (**H**) Overlay demonstrating the colocalisation of CD34 and TGF‐β1 (yellow) in telocytes. Original magnification was 200×, scale bar represents 50 μm.

**Figure 6:**
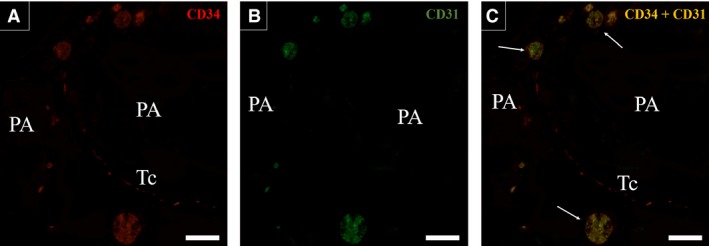
Double immunofluorescence assays for CD34/CD31 in the interalveolar region of a histological section of Mongolian gerbil prostate on P30. (**A**) CD34 labelling (red) is verified in blood vessels and in a telopode of a telocyte that displays a monoliform aspect. (**B**) CD31 immunolabelling is seen in blood vessels but not in the telocyte (**C**) Overlay in which is possible to observe that blood vessels demonstrate the colocalisation of CD34 and CD31while the telocyte is exclusively CD34 (red) positive. Original magnification of 200×, scale bar represents 50 μm. PA (prostatic alveolus), Tc (telocyte), arrows (blood vessels).

The double immunofluorescence assays for CD34/ERβ and CD34/TGF‐β1 in telocytes after 96 hrs of primary culture showed the presence of CD34‐positive cells and nuclear labelling for ERβ (Fig. 5A and D). CD34‐positive cells in the telocyte culture also showed labelling for TGF‐β1. The presence of ERβ in the prostatic telocytes indicates that these cells possibly respond to a late pathway activated in prostatic development that leads to a reduction of proliferative activity and stimulates epithelial differentiation. Double immunofluorescence assays were also performed for CD34/CD31 to distinguish telocytes from blood vessels in the interacinar region, as both are CD34 positive. It was verified that blood vessels are positive for CD34 and CD31, and telocytes are exclusively CD34 positive, as well as telocytes show long CD34‐positive telopodes demonstrating a morphology different from blood vessels CD34 staining (Fig. 6A–C).

**Figure 7:**
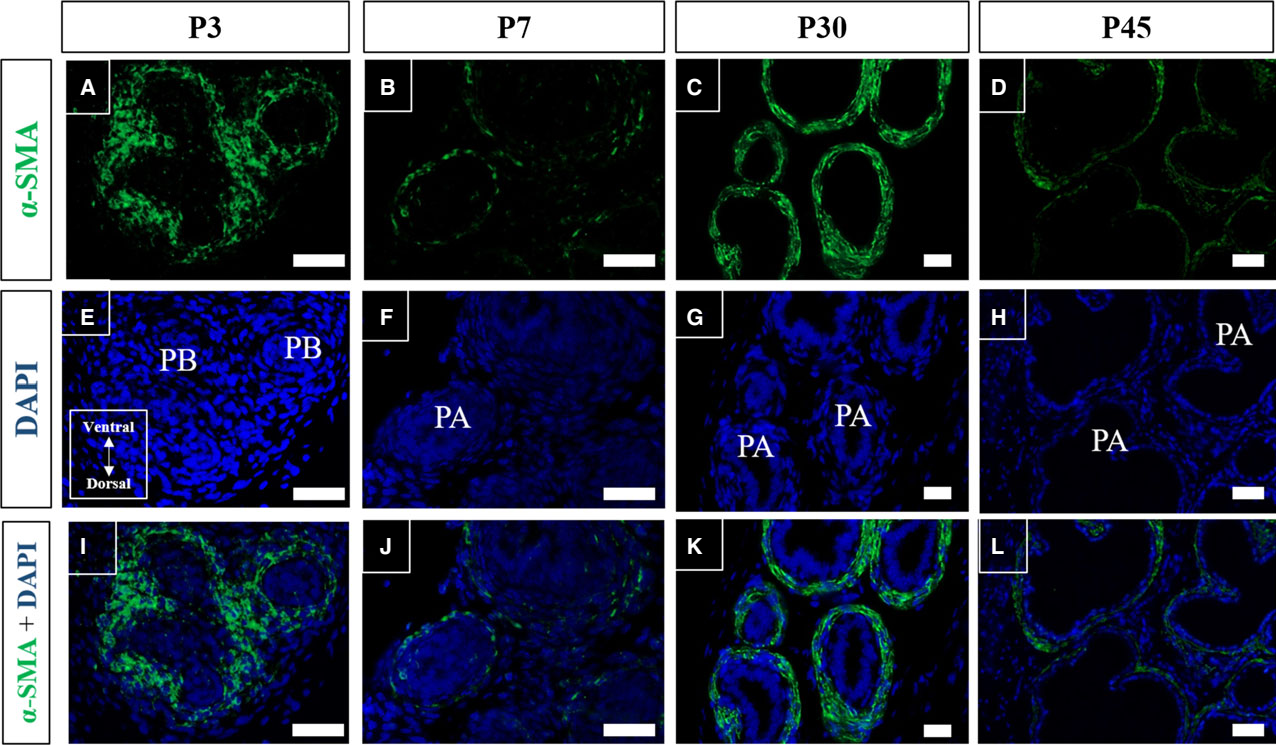
Immunofluorescence assays for α‐SMA in histological sections of Mongolian gerbil prostate on different days of postnatal development. (**A**,** E** and **I**) α‐SMA labelling (green) is observed on the periphery of prostatic branches on P3 in stromal progenitor cells of periductal smooth muscle. (**B**,** F** and **J**) Labelling for α‐SMA in periductal smooth muscle surrounding developing alveoli on P7. (**C**,** G** and **K**) The periductal smooth muscle is already differentiated by P30. (**D**,** H** and **L**) Alveoli lumen expands and periductal smooth muscle has reached its characteristic conformation by P30. Original magnification of 200×, scale bar represents 50 μm. PB, prostatic branching; PA, developing prostatic alveoli.

Immunofluorescence assays for α‐SMA in the histological sections of Mongolian gerbil prostate on different days of postnatal development were performed to characterise the developmental progression of the periductal and perialveolar muscles. Labelling for α‐SMA (green) was observed at the periphery of the prostatic branches on smooth muscle progenitor cells in prostates collected at P3 (Fig. 7A, E and I). Labelling of α‐SMA was observed in the developing periductal smooth muscle in the prostate alveoli on P7 (Fig. 7B, F and J). By P30, the periductal smooth muscle had already differentiated (Fig. 7C, G and K), and the lumen of the alveoli had expanded and periductal smooth muscle showed its characteristic conformation (Fig. 7D, H and L).

**Figure 8:**
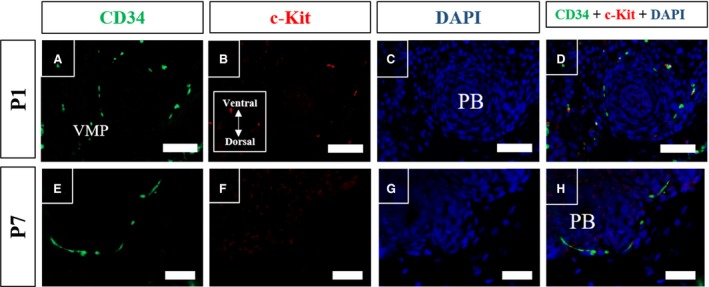
Immunofluorescence assays for CD34 (green) and c‐kit (Red) in histological sections of Mongolian gerbil prostate of early postnatal development. (**A**–**D**) In the perinatal period (P1), labelling of CD34 and of c‐Kit is dispersed in the VMP. (E‐H) Labelling of CD34 progresses in the region surrounding prostatic budding on P7, while c‐Kit labelling remains disperse along the VMP. Original magnification was 200×, scale bar represents 50 μm.VMP, ventral mesenchymal pad; PB, prostatic branching; St, prostate stroma; PA, developing prostate alveoli.

**Figure 9:**
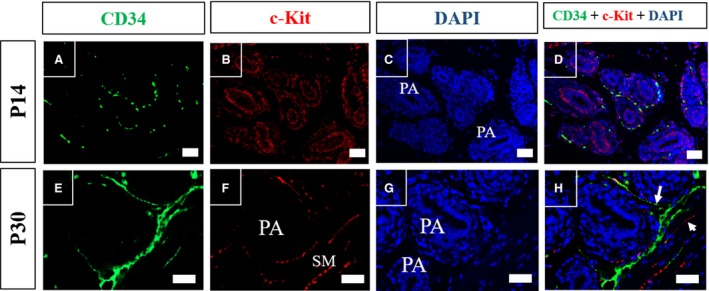
Immunofluorescence assays for CD34 (green) and c‐kit (Red) in histological sections of Mongolian gerbil prostate of late postnatal development. (**A**–**D**) Immunolabelling for CD34 is observed surrounding the periductal/perialveolar region on P14, inside this region immunolabelling for c‐Kit is verified involving the developing periductal smooth muscle layer. There is also a colocalisation of these factors at the periphery of the periductal/alveolar region. (**E**–**H**) On P30, there is immunolabelling for CD34 in the stroma that surrounds the periductal smooth muscle and between the prostatic alveoli, whereas the c‐Kit is verified in the periductal and periurethral smooth muscles. Colocalisation of CD34 and c‐kit is observed in some stromal cells in the periductal region during early prostate postnatal development, and it is also verified in some stromal cells of the perialveolar region on P30. These factors do not colocalize on the stromal cells of the adjacent region of the periurethral smooth muscle, which are only positive for CD34. C‐Kit‐positive stromal cells are verified in the periurethral smooth muscle, along with few CD34‐positive stromal cells or cells that show colocalisation of both factors. Original magnification was 200×, scale bar represents 50 μm. SM, periurethral smooth muscle; PB, prostatic budding/branching; PA, developing prostatic alveoli. The arrow indicates the CD34‐positive cells at the periphery of periurethral smooth muscle and in the interalveolar region. The arrowhead indicates c‐kit‐positive cells in the periurethral smooth muscle.

To evaluate the labelling pattern of the two main markers used for telocyte characterisation, immunofluorescence assays were performed for CD34 and c‐kit in histological sections of the prostate of Mongolian gerbil on different days of postnatal development. The immunolabelling for CD34 was found to be dispersed in the early postnatal period, and progressed to concentrate in the periphery of the differentiating alveoli, coinciding with smooth muscle differentiation in the perialveolar region and later it was verified in the region between alveoli and in the region surrounding the periurethral smooth muscle(Figs 8A and E; 9A and E). The immunolabelling for c‐kit was found disperse initially, and was arranged adjacent to the prostatic epithelium of the developing alveoli on P14 and P30, involving the periductal smooth muscle, as well as it was verified in the periurethral smooth muscle during this period (Figs 8B and F; 9B and F). Colocalisation of CD34 and c‐kit was observed in the periductal region of some cells during early postnatal development of the prostate, and on P30 it was also seen in the perialveolar region. However, these factors do not colocalise on the stromal cells in the adjacent region of the periurethral musculature, as these cells were only positive for CD34. In the periurethral smooth muscle, most immunolabelled cells were c‐kit positive, and the minority were CD34 positive or showed colocalisation of c‐kit and CD34 (Fig. 9E–H).

**Figure 10:**
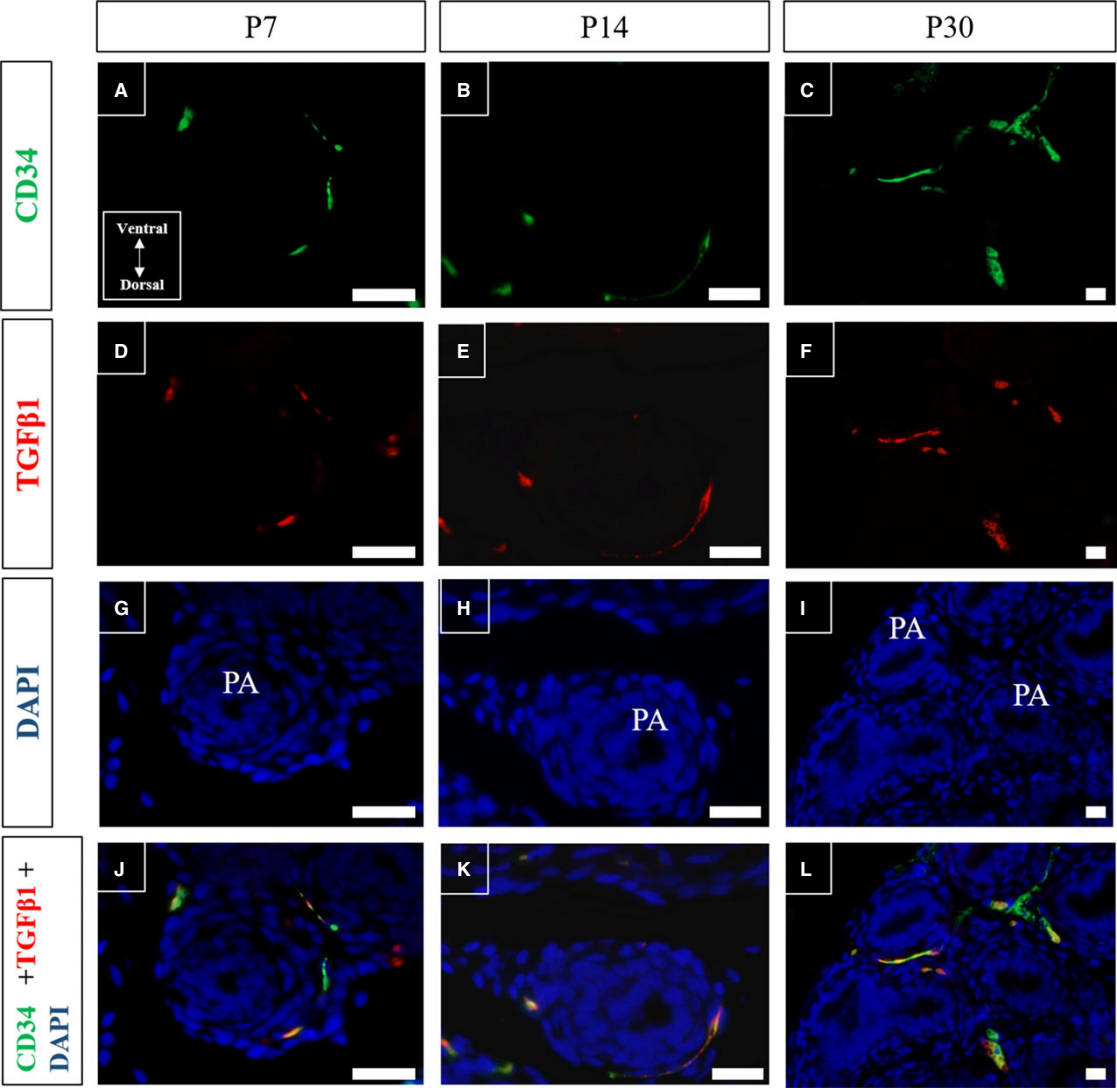
Immunofluorescence assays for CD34 (green) and TGF‐β1 (red) in histological sections of Mongolian gerbil prostate on different days of postnatal development. (**A**–**C**) Telocytes progressively acquire their characteristic phenotype with the formation of long CD34‐positive telopodes, observed at the periphery of the developing prostatic alveoli on P14. By P30, telocytes had formed a network that had spread to the interalveolar region. (**D**–**F**) Labelling for TGF‐β1 focussed mainly in the periductal/perialveolar region during prostatic postnatal differentiation. (**G**–**I**) Labelling for DAPI (blue). (**J**–**L**) Colocalised labelling of CD34 and TGF‐β1 in the periductal/perialveolar region during postnatal differentiation of the prostate, whereas only limited colocalisation can be observed in the interalveolar region. Original magnification was 200×, scale bar represents 50 μm. PB, prostatic branching; PA, developing prostatic alveoli.

The possible relationship between telocytes and smooth muscle differentiation was evaluated using immunofluorescence assays for CD34 and TGF‐β1 in histological sections of Mongolian gerbil prostate on different days of postnatal development. The telocytes progressively acquired their characteristic phenotype, with the formation of long CD34‐positive telopodes, which were observed on the periphery of the developing prostate alveoli on P14. By P30, the telocytes had formed a network that spread to the interalveolar region (Fig. 10A–C). The immunolabelling of TGF‐β1 was concentrated in the periductal/perialveolar region during prostatic postnatal differentiation (Fig. 10D–F). Colocalisation of CD34 and TGF‐β1 labelling was found in the stromal cells of the periductal/perialveolar region throughout postnatal prostate differentiation, whereas a smaller degree of colocalisation was observed in the interalveolar region (Fig. 10J–L).

**Figure 11:**
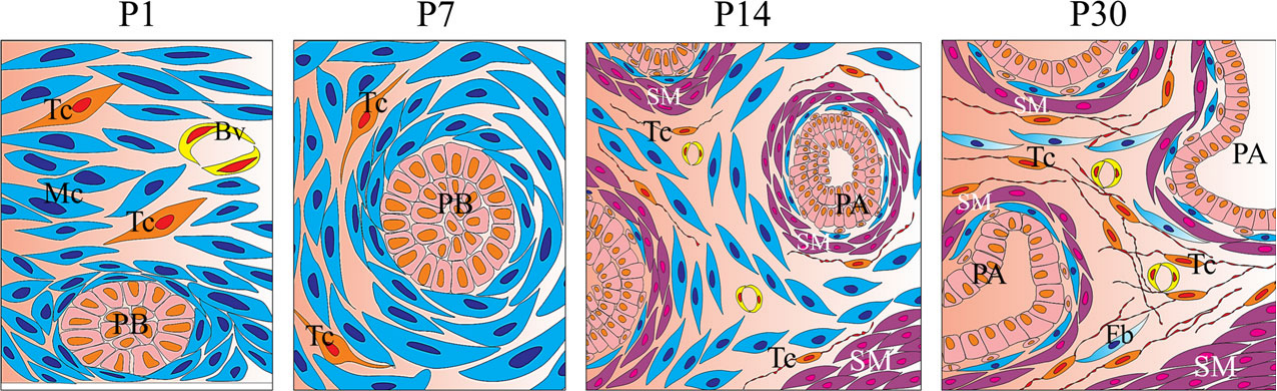
Schematic depicting prostate development and the possible role of telocytes. On P1, telocyte progenitor cells are dispersed throughout the stroma. By P7, telocytes are present at the periphery of the cells that give rise to the perialveolar smooth muscle, followed by rapid development, in which the phenotype of telocytes is similar to mature telocytes. On P30, telocytes surround the perialveolar muscle, as well as being found in the interalveolar region, separating clusters of alveoli from each other and acting as a barrier between alveoli and the periurethral smooth muscle. Tc, telocytes; PB, prostate budding; Mc, mesenchymal cell; Bv, blood vessel; SM, perialveolar smooth muscle; SM, periurethral smooth muscle; PA, developing prostate alveoli; Fb, fibroblast.

The data from the immunofluorescence assays have been represented in a schematic (Fig. 11) that depicts prostate development and the possible role of telocytes.

## Discussion

Prostate telocytes are thought to perform a supportive role in the contraction of periductal smooth muscle and in stromal compartmentalisation 8. We have demonstrated that telocytes in cell culture are ERβ positive, suggesting sensitivity to oestrogens, which are associated with differentiation of prostatic epithelium *via* the ERβ signalling pathway, and with reduced stromal proliferation 32, 33, 34, thus, the telocytes are possibly involved in the developmental stage of morphological differentiation of the prostatic alveoli and ducts. Corroborating this hypothesis, we also found that telocytes produce TGF‐β1 in primary culture, which is an antiproliferative paracrine factor involved in differentiation of the periductal and perialveolar smooth muscle 35, 36.

In prostate tissue, our ultrastructural data demonstrated that, in the neonates, the telocytes are not yet verified in the periphery of the prostatic budding, they are verified only in the interstitium. At the end of the first week of postnatal life (P7), cells with thick cytoplasmic processes are seen interspersed in the periductal smooth muscle, possibly consisting of undifferentiated telocytes, such cells are surrounded by differentiated telocytes. On the onset of prematuration (P45), telocytes are seen surrounding the prostatic alveoli, cells resembling telocytes with thick cytoplasmic processes are also observed surrounding the prostatic alveoli, such data may indicate a possible supportive role of prostate telocytes on periductal smooth muscle differentiation.

In this sense, the immunofluorescence assays for α‐Actin and CD34 corroborate the possible role of telocytes on smooth muscle differentiation at the tissue level, showing that these cells differentiate simultaneously with muscles, in which α‐Actin immunolabelling initially disperse around the prostatic buds becomes concentrated on the thin layers of the differentiated perialveolar smooth muscle, at the same time, the immunolabelling for CD34 is verified surrounding the periductal/alveolar region in which smooth muscle differentiates. Moreover, telocytes in the prostatic tissue were also TGF‐β1 positive, which validates the data obtained from cell culture, and supports the possible role of prostatic telocytes in the morphogenesis of the gland, particularly in the differentiation of periductal smooth muscle *via* secretion of TGF‐β1.

In addition to playing a direct role in prostate morphogenesis *via* paracrine signalling, prostatic telocytes may also contribute to prostate morphogenesis by stromal compartmentalisation. This would result in the formation of separate stromal microenvironments, as previously observed in the myocardium, in which telocytes are involved in the development of cardiomyocytes, and are spread among cardiomyocyte groups, thereby compartmentalising the myocardium 37.

Our data are suggestive of the formation of telocyte networks between the perinatal period and P30, which form potential barriers between stromal compartments in the prostate and occupy portions of the interalveolar region. Such telocyte networks separate the alveoli groups, as well as separating the interalveolar region from the periurethral smooth muscle, which is an important structure for the establishment of prostate tissue architecture, and consequently, its function. In addition, large deposition of collagen fibrils at the periphery of telopodes may indicate that telocytes, such as fibroblasts, may act directly in the production of extracellular matrix components. In terms of prostatic morphogenesis, our ultrastructural and immunofluorescence data indicate that the telocytes differentiate at different times among prostatic compartments, being present in the interstitium before they are present at the periphery of the prostatic budding and that such cells differentiate together with the smooth muscle cells in the periductal region, at the same time as telopode networks expand through the interalveolar region.

Immunolabelling for CD34 and c‐Kit was performed for the detection of prostate telocytes, despite both being telocytes markers, the overlap of these factors in the prostate tissue varies along the developmental time and among the prostate regions. The existence of interstitial cells that were exclusively positive for c‐Kit or CD34 labelling may indicate functional differences between such cells. C‐kit had been established as a marker for interstitial cells, the ICCs, since before the characterization of telocytes 38. The primary function assigned to these cells was a pacemaker function in the contraction of smooth muscle 19, 20. C‐kit is a receptor that binds SCF, resulting in the mobilisation of calcium *via* a src family kinase and PI3K 22. In addition to its functional importance, c‐Kit is also essential for the differentiation of ICCs 39, 40,as CD34 is a typical marker for a wide variety of blood progenitor cells 41, and more generally, a marker of undifferentiated cells in various tissues 42.

It has previously been proposed that CD34‐positive fibroblast‐like ICCs cells were progenitors of c‐kit‐positive ICCs in the intestine 43, 44. Our data point to the presence of CD34‐positive cells surrounding prostatic budding on the early prostate development, later CD34/c‐kit‐positive cells and c‐kit‐positive cells are verified in this region, while CD34‐positive cells form a network of telopodes in the interalveolar region. These findings could indicate the differentiation of CD34‐positive cells into c‐Kit‐positive cells, with the intermediate phase including cells that are positive for both CD34 and c‐Kit in the perialveolar stroma, and the existence of CD34‐positive cells that perform other functions as supporting stromal organisation in the interalveolar region. In the same sense, our ultrastructural data pointed to the existence of cells with thick cytoplasmic processes at the periphery of the periductal smooth muscle on P45, such cells possess triangular cell bodies and may consist of cells similar to ICCs. These cells were described in the prostate prior to the description of prostatic telocytes and to them were assigned the generic name of interstitial cajal‐like cells (ICLCs) 45. The characterization of telocytes was useful to avoid multiple ambiguous terminologies for CD34‐positive fibroblast‐like cells found in various organs, our data confirm the existence of fibroblast‐like CD34 and CD34/c‐Kit‐positive cells, consisting of prostatic telocytes, however, the data also point to the existence of fibroblast‐like cells c‐Kit positive and CD34 negative 9, 10, 11, 12, 13, 14, 15, 16, 17, 18, to which the canonical definition of telocytes is not applied. Moreover, in structural terms we obtain some evidence of the existence fibroblast‐like cells that possess shorter and thicker cytoplasmic process at the periphery of the developing perialveolar smooth muscle, in which c‐Kit‐positive/CD34‐negative cells are verified.

Moreover, the periurethral smooth muscle that differentiates earlier than periductal/alveolar smooth muscle showed predominantly c‐Kit‐positive cells, with small interspersed populations of CD34‐positive cells and positive cells for both factors. This further supports the possible cell differentiation of telocytes into c‐Kit‐positive fibroblast‐like cells similar to ICCs. These evidence are consistent with the data on the differentiation of ICCs, in which is demonstrated the existence of CD34‐positive progenitors, which give rise to CD34 and c‐Kit‐positive cells and, finally, differentiate into exclusively c‐Kit‐positive mature ICCs 44, 46, 47.

The interalveolar region contained predominantly CD34‐positive cells on P30, with the formation of networks that separate the clusters of alveoli from each other and separate clusters of alveoli from the periurethral smooth muscle, thus attributing to the exclusively CD34‐positive interstitial cells uniquely a role of ICCs progenitor cells is a limited proposition, in view of the evidence that these cells possess many other functions in the tissue organisation and functionality in various organs 8, 10, 11, 12, 17, 18, 24, 25. CD34‐positive telocytes that do not express c‐Kit may also perform a pacemaker function 48, 49 *via* c‐Kit‐independent pathways, such as the SK3 pathway, by inhibiting electrical activity. This is unlike the stimulating pathways activated by c‐Kit 20, 23, and therefore may play a complementary role to the pacemaker activity exerted by c‐Kit‐positive telocytes or ICCs.

In general terms, our work supports that telocytes play a role in prostate morphogenesis and tissue compartmentalisation, as already demonstrated for myocardium 50, and these cells possibly have a supportive role in the differentiation of periductal/alveolar smooth muscle, as well as contributing by means of a telopode network to tissue compartmentalization of the prostatic stroma in different microenvironments and possibly producing components of the extracellular matrix. In addition, our data point to the existence of fibroblast‐like cells in the prostate that are c‐Kit positive and CD34 negative, which escape the typical immunolabelling profile of telocytes (CD34 or CD34/c‐Kit positive) 9, 10, 11, 12, 13, 14, 15, 16, 17 And in the face of the immunolabelling profile of these factors during prostate development, in addition to some ultrastructural evidence, it is suggested that some telocytes (CD34 or CD34/c‐Kit positive) present at the periphery of the developing smooth muscle of alveoli could differentiate into c‐Kit‐positive and CD34‐negative fibroblast‐like cells that resemble ICCs. However, further studies will be needed to test this hypothesis.

## Conflicts of interest

The authors declare that there are no conflicts of interest associated with this work.
